# The Progress and Prospects of Putative Biomarkers for Liver Cancer Stem Cells in Hepatocellular Carcinoma

**DOI:** 10.1155/2016/7614971

**Published:** 2016-08-17

**Authors:** Yan Xiang, Ting Yang, Bing-yao Pang, Ying Zhu, Yong-ning Liu

**Affiliations:** ^1^Department of Infectious Disease, The First Affiliated Hospital of Dalian Medical University, Dalian, Liaoning 116011, China; ^2^Department of Nursing, The First Affiliated Hospital of Dalian Medical University, Dalian, Liaoning 116011, China

## Abstract

Accumulating evidence suggests that hepatocellular carcinoma (HCC) is organized by liver cancer stem cells (LCSCs), which are a subset of cells with “stem-like” characteristics. Identification of the LCSCs is a fundamental and important problem in HCC research. LCSCs have been investigated by various stem cell biomarkers. There is still lack of consensus regarding the existence of a “global” marker for LCSCs in HCC. In this review article, we summarize the progress and prospects of putative biomarkers for LCSCs in the past decades, which is essential to develop future therapies targeting CSCs and to predict prognosis and curative effect of these therapies.

## 1. Introduction

Hepatocellular carcinoma (HCC) is one of the most common tumors, which is the third leading cause of cancer-related death worldwide and increases the global disease burden [1]. At present, the details of the mechanism responsible for HCC formation and maintenance are still unclear. HCC is a major health issue because of its poor prognosis and few available treatment options [2]. A recent subtype of HCC suggested that HCC expresses stemness-related markers, in which a portion of tumor cells (>5%) express stem/progenitor cell markers. At present, it is widely accepted that CSCs participate in the courses of tumor initiation, progression, metastasis, and relapse [3, 4] Therefore, identification of CSCs and CSC-related therapeutic targets is necessary for improving HCC treatment outcome [5]. As a consequence, by the new and ongoing research continuing, the HCC biomarker discovery field is rapidly expanding, which proposes a fast growing list of biomarker candidates. Numerous LCSC biomarkers have been identified including cell surface or membranous proteins (calcium channel a2d1 isoform 5, CD133, CD90, CD44, CD47, CD15, CD24, CD13, CXCR4, EpCAM, ABC transporters, DLK1, Nope, and DCLK1), cytoplasmic proteins (OV6, nestin, Musashi-1, ALDH, and CK19), or nuclear proteins (SOX2, SOX9, Oct3/4, and Nanog). This review summarizes recent discoveries about biomarkers relevant to LCSCs recognition and hopes these markers may contribute to diagnosis and prognosis prediction in patients with HCC, as well as improving HCC patients' survival.

## 2. Markers on the Cell Surface or Membranous Proteins

### 2.1. CD133 (Prominin-1)

Human CD133, a 5-transmembrane single-chain glycoprotein, pertains to the prominin family containing two large extracellular and two small intracellular loops, respectively [6–8]. It was originally recognized as a cell surface antigen that appeared on CD34^+^ hematopoietic stem cells [6]. It is an important CSCs surface marker which has been documented in various tissues including the cancer of liver [9–11].

By investigating CD133 in 3 hepatocyte cell lines, Suetsugu and his colleagues [12] found that CD133 was only expressed in the Huh7 cells. And they first reported that CD133^+^ HCC cells represented a potential CSC subpopulation in HCC. Piao et al. [11] found that CD133^+^ cells accounted for 65% of Huh7 cells. CD133^+^ cells had a greater colony-forming efficiency and higher proliferative and greater ability to form tumor in immunodeficient mice by Ma et al. [13]. The similar results were found by Yin et al. [14] in a CD133^+^ portion isolated from the HCC cell line SMMC-7721. During early liver restoration the expression of prominin-1, the homolog of human CD133 in mice, was found to be significantly upregulated [15]. Rountree et al. [16] found that CD133^+^CD45^−^ cells had CSC characteristics. At the stage of primary carcinoma formation, they discovered that the CD133^+^CD45^−^ cells from chronic liver disease represented a bipotent liver stem cell population. A research by Zhu et al. [17] revealed that CD133^+^CD44^+^ cells were more tumorigenic and chemoresistant when exposed to cytotoxic drugs such as doxorubicin and vincristine. Recently, a meta-analysis comprising 2592 HCC patients by Zhong et al. [18] found that the high expression of CD133 was significantly associated with a range of clinicopathological features, such as low tumor stage, advanced tumor stage, vascular invasion, vascular thrombosis, and poorer survival outcome. Above all, CD133 is supported as a marker of liver cancer stem/progenitor cells.

Compared with CD133^−^ counterparts, further studies by Ma et al. [19] showed that their CD133^+^ cells were more resistant to conventional chemotherapeutic drugs, including doxorubicin and 5-fluorouracil, by preferential activation of the Akt/PKB and Bcl-2 survival pathway. Piao et al. [11] investigated that the xenograft model exhibited increased tumor formation in nude mice which irradiated CD133^+^ cell injected by activation the MAPK/ERK survival pathway, which suggested that CD133 cells were conductive to radioresistance in HCC. Using a JNK specific inhibitor, Hagiwara et al. [20] found that the xenografted CD133^+^ cells could be reduced in athymic mice, which identified that the therapeutic reaction of HCC patients to sorafenib was negatively correlated with CD133 expression and JNK pathway activity. In conclusion, these studies demonstrate that liver cancer stem cells express CD133 to escape conventional chemotherapeutic agents and radiation exposure in HCC through activation of association survival pathway.

Several other studies supported the therapeutic potential through targeting CD133 cells. In HCC, the expression of Ikaros decrease was relative to poor survival and the study by Zhang et al. [21] showed that Ikaros exerted an antitumor effect through interacting with CtBP as a transcriptional repressor complex that directly binded to the CD133 P1 promoter so that the CSCs properties of HCC cells were inhibited. Lin et al. [22] found that ursolic acid chalcone (UAC) generated a decrease in self-renewal capability and an increase in sensitivity to doxorubicin and vincristine drugs to inhibit the expression of CD133^+^ in a dose- and time-dependent manner in PLC/PRF/5 and Huh7 HCC cells, which could be a potent therapeutic agent to target differentiation of CSC in HCC. In the peritoneal cavity of nude mice, oncolytic measles virus targeting CD133^+^ HCC cells has shown antineoplastic effect on HCC growing subcutaneously or multifocally [23]. Knockdown of 14-3-3*ζ* decreased cell viability and the number of spheres besides upregulating the level of proapoptotic protein in CD133^+^ liver CSCs after *γ*-irradiation (IR) [24]. The overexpression of IGF2BP3 enriched the CD133^+^ CSC subgroup in HCC, which enhanced tumor sphere formation and suppressed the cytotoxic effects of sorafenib and doxorubicin. A recent study by Li et al. [25] found that a derivative of isocorydine (d-ICD) inhibited HCC cells growth, especially among the CD133^+^ subpopulation, and rendered that HCC cells are more sensitive to sorafenib treatment. These results highlight the importance of targeting to silence the CD133^+^ liver CSCs in order to improve treatment outcome of HCC.

### 2.2. CD90

CD90 (Thy-1) is a heavily glycosylated, 25–37 kDa glycosylphosphatidylinositol- (GPI-) anchored cell surface protein expressed on many cell types, including T cells, thymocytes, neurons, endothelial cells, and fibroblasts. CD90 is an important regulator of cell-cell and cell-matrix interactions, playing an important role in metastasis, inflammation, and fibrosis [26].

Recently, CD90 has also received attention as a CSC marker in various tumor cells, including hepatic stem cells. As a potential marker, CD90 depicted CSCs serially from HCC cell lines, human hepatocellular carcinoma specimens, and blood samples [27]. In all of the tumor samples and 90% of the blood specimens from HCC patients the CD45^−^CD90^+^ cells were detected, while there was a very low number (0%–0.05%) in the normal, cirrhotic, and parallel nonneoplastic livers by flow cytometry [27]. These indicate that CD90 expression increases during tumor formation and generates tumor nodules. Therefore, CD90 may be considered to be a marker for LCSCs.

Yang et al. [27, 28] showed that the CD90^+^ cells from HCC cell lines revealed higher oncogenicity compared with the CD90^−^ cells. Liu et al. [29] indicated that CD90 might be more sensitive in predicting poor differentiation in HCC. Cheng et al. [30] found that CD90 probably regulated the invasion and migration of liver cancer. Their results showed that higher expression of CD90 occurred in neoplastic hepatic tissues and poorly differentiated liver tumors when compared with control tissues and well-differentiated ones. Further, HepG2 cells which were transfected with pReceiver-M29/thy-1 detected higher expression of CD90 than E-cadherin (a marker of invasion and migration). In another research [31] they suggested that the upregulation of CD90 promoted the proliferation and inhibited apoptosis of HepG2 cells, and these processes were regulated by Wnt/*β*-catenin signaling pathway. A research [32] revealed that the existence of CD90^+^ cells was relative to a high incidence of distant organ metastasis. Chen et al. [33] found that the signal axis of CD90-integrin-mTOR/AMPK-CD133 was critical for promoting liver carcinogenesis. These indicate that CD90 plays a crucial role in HCC proliferation, invasion, migration, and metastasis.

Recently, an increasing number of researches have indicated that the downregulation of CD90 may be a potential therapeutic target in HCC. In Liu et al.'s [29] study they found that CD90 had specificity of 91.9% for HCC and sensibility of 48.22% in predicting low differentiation, which demonstrated that CD90 was a promising target for patients with HCC. Compared with the CD90^+^CD44^−^ counterparts, the CD90^+^CD44^+^ cells displayed a more aggressive phenotype and formed metastatic lesions in the lung of immunodeficient mice. This indicated that the formation of local and metastatic tumor nodules could be prevented by knocking CD44 in the CD90^+^ cells [28]. By using aspirin as an inhibitor of the Wnt pathway in the treatment of HepG2 cells which were transfected with the pReceiver-M29/thy-1 expression vector, Cheng et al. [31] conducted detailed observations of apoptosis in HepG2 cells as well as the differential expression of *β*-catenin, cyclin D1 (a downstream transcription factor of Wnt/*β*-catenin signaling pathway), and CD90. They detected an increasing apoptosis rate in the HepG2 cells and downregulating expression of the three proteins. Chen et al. [33] also suggested that molecules which inhibited the signal axis of CD90-integrin-mTOR/AMPK-CD133, including OSU-CG5 and other inhibitors, maybe served as potential novel cancer therapeutic targets in liver cancer.

### 2.3. CD44

CD44, which acts mainly as receptors for hyaluronan, is a member of the family of transmembrane glycoproteins. It is involved in mediating adhesion between cells and the extracellular matrix, lymphocyte activation, and homing and plays a dominant role in invasion and metastasis of cancer [34, 35]. It has been identified as a CSC biomarker in breast, colorectal, pancreatic, hepatocellular, and gastric carcinoma [36–40]. Hirohashi et al. [41] suggested that the expression of CD44 in HCC was related to a higher extrahepatic metastasis rate and a lower frequency of survival. Furthermore, Cogliati et al. [42] used immunohistochemical analysis to investigate stem/progenitor cells in 13 HCC and 7 CC archived specimens. They found that 61.6% of HCC samples appeared to be immature CD44^+^ hepatocytes, but only two samples presented CD133^+^ cells. Recently, a research by Fernando et al. [43] found that the resistance to sorafenib could be induced by TGF-*β* treatment of long term-cultured PLC/PRF/5 liver cancer cells. Further analysis found that epithelial-to-mesenchymal transition (EMT) which was characterized by vimentin protein expression could be induced by the expression of CD44, but the drug resistance was directly proportional to the expression of CD44^+^ cells. Moreover, sorafenib repeated treatment could enrich CD44^+^ cells. Therefore, CD44 might be a potential molecular marker of LCSCs.

Instead of being an independent marker of LCSCs in HCC, CD44 appears to be more useful combined with other markers. Many studies suggested that the double-positive for CD44 and CD133 or CD90 could better define LCSCs. Zhu et al. [17] found that the coexpression of the cell surface biomarkers CD133 and CD44 could precisely define the LCSCs phenotype. They suggested that CD133^+^CD44^+^ HCC cells exhibited stem cell properties, such as extensive proliferation, self-renewal, and differentiation into the majority of cancer cells. In nude mice, CD133^+^CD44^+^ cells showed higher tumorigenic ability than CD133^+^CD44^−^ counterparts. Moreover, they also observed that the CD133^+^CD44^+^ cells in HCC cell lines exhibited the advantage of expressing some stem cell-associated genes such as *β*-catenin and BMI-1 and were more resistant to multiple chemotherapeutics such as doxorubicin and vincristine on account of the upregulation of ATP-binding cassette (ABC) super family transporters (ABCB1, ABCC1, and ABCG2). Hou et al. [44] showed that the CD133^+^CD44^+^ cells were the initial cells that produced metastasis to the lung and liver in immunodeficient mice. Analyses of human liver samples revealed that CD133^+^CD44^+^ liver cancer cells were related to metastasis to the liver portal vein. Compared with CD90^+^CD44^−^ counterparts, a higher capacity of inducing lung metastasis has been demonstrated in CD90^+^CD44^+^ cells, indicating that CD44 could be used as a marker further to specify CD90^+^ CSCs with a higher metastatic potential. It was first reported by Yang et al. that CD90^+^CD44^+^ liver cancer cells were more aggressive [27, 28]. Knockdown of CD44 by a neutralizing antibody induced apoptosis of CD90^+^ cells, which suppressed engraftment and inhibited lung metastasis in mice. A 22-fold increase in CD44^+^CD90^+^ cells was found by Thompson et al. [45] after irradiation of N1S1 rat liver cancer cells, in which CD44^+^ cells increased, while CD90^+^ cells did not change.

### 2.4. CD24

CD24 is a small, heavily glycosylated mucin-like cell surface protein anchored to the membrane by phosphatidylinositol and has been shown to be overexpressed in stem/progenitor cells and has been linked to CSCs derived from breast, colon, ovarian, pancreatic, and hepatocellular carcinomas [46–51]. Huang and Hsu [52] suggested that the CD24 gene expression appeared to be common in HCC and may be used as an early but not prognostic marker for malignant transformation of hepatocytes. They first cloned the full-length CD24 from the cDNA sequence of human HCC and revealed that there was a strong correlation between CD24 mRNA overexpression, p53 gene mutation in HCC, and poorly differentiated HCC. CD24 is a LCSC biomarker which is closely relative to maintenance, self-renewal, metastasis, differentiation, chemoresistance, and recurrence of HCC [51, 53–56]. Lee et al. [53] found that CD24 was a functional liver T-ICs marker that derived T-IC genesis through STAT3-mediated NANOG regulation by lentiviral-based knockdown approach. Liu et al. [57] revealed that Twist2 increased LCSC self-renewal in a CD24-dependent manner and suggested that Twist2-CD24-STAT3-Nanog pathway may have played a dominant role in regulating LCSC self-renewal. Li et al. [56] demonstrated that targeting genetic variation of CD24 obviously reduced the sizes of primary HCC in the HBV transgenic mice, which suggested that the mutation of CD24 may be an enormous determinant for the outcome of chronic HBV infection.

Recently, CD24 has become a novel targeted therapeutic in improving the outcome of HCC patients. Zheng et al. [58] revealed that NDRG2 (N-myc downstream-regulated gene 2) upregulated displayed anticancer activity by decreasing CD24, a molecule that mediated cell-cell interaction, tumor proliferation, and adhesion. By dose- and time-dependent manners in HCC cells it has been found that baicalein remarkably decreased the expression of c-Myc, a critical regulator of cell growth, apoptosis, and cellular transformation. Recently, a research by Han et al. [59] demonstrated the efficient antitumor effects of baicalein on HCC cells and indicated that baicalein suppressed cell proliferation and cell survival through downregulation of CD24. He et al. [60] found that G7mAb and G7S were assembled in CD24^+^ Huh7 HCC xenograft tissue via specific binding to CD24 in vivo by near infrared fluorescence imaging, which exhibited tumor targeted therapeutic and diagnostic potentials in vitro and in vivo.

### 2.5. CD13 (Aminopeptidase N)

CD13, also known as aminopeptidase N, is a membrane glycoprotein that exhibits CSC characteristics and plays important roles in cancer progression such as cells proliferation, formation of cellular clusters in cancer foci, and the ability to survive during treatment [61–64]. Christ et al. [65] revealed that CD13^+^ cells played a dominant role in the G0 phase of the cell cycle and typically formed cellular clusters in cancer lesion. Recently, CD13 has been identified as a novel cell surface marker for semiquiescent CSCs in human HCC cell lines and clinical specimens, and CD13^+^ CSCs were relative to a hypoxic marker in clinical HCC sample. These suggest that CD13^+^ CSCs have a dominant role in tumor proliferation and resistance to antitumor therapy in HCC [66].

By adopting Hoechst dye exclusion approach, Haraguchi et al. [64] explored that CD13 expressed side population as a semiquiescent CSC subpopulation in human HCC. The CD13^+^ cells were equipped with a high tumorigenic potential in NOD/SCID mice and also displayed resistance to chemotherapeutic drugs, doxorubicin and 5-fluorouracil. These results were attributed to the low levels of intracellular reactive oxygen species (ROS), which protected the cells from DNA damage and apoptosis after genotoxic chemo/radiation stress. They further demonstrated in mouse xenograft models that combination of CD13 inhibitor (ubenimex) with the genotoxic chemotherapeutic fluorouracil (5-FU) resulted in drastic tumor regression compared with either agent alone. 5-FU alone inhibited proliferative CD90^+^ CSCs and generated semiquiescent CD13^+^ CSCs. CD13 inhibitor (ubenimex) suppressed the self-renewing and tumor-initiating potential of dormant CSCs. Thus, it suggested that CD13 inhibitor integrated with ROS-inducing chemo/radiation therapy could improve the outcome of the HCC. Martin-Padura et al. [67] revealed that CD13^+^ cells were in minimal residual disease (MRD) via orthotopic HCC mouse models by injection of human AFP and/or luciferase-expressing HCC cell lines and primary HCC cells, and this also suggested that targeting dormant CSCs was critical for treatment success. Furthermore, a research by Wang et al. [68] found that both Notch and Wnt/*β*-catenin signaling pathways played vital roles in increasing the stemness characteristics of LCSCs (such as CD90, CD24, CD13, and CD133). Ectopic expression of Notch1 intracellular domain (NICD) with lentivirus N1ShRNA-Notch1 in LCSCs weakened *β*-catenin/TCF dependent luciferase activity significantly. In addition, there was a nonproteasome mediated feedback loop between Notch1 and Wnt/*β*-catenin signaling in LCSCs. They speculated that the critical role of Notch and Wnt/*β*-catenin signaling pathways in LCSCs might provide an attractive therapeutic strategy against HCC.

### 2.6. EpCAM (CD326)

EpCAM, also known as CD326, is a homophilic, Ca^2+^-independent cell-cell adhesion molecule expressed in many human epithelial tissues. EpCAM is expressed in nontumor liver tissues, such as fetal hepatoblasts, bile duct epithelial cells, proliferating bile ductules, LCSCs, and premalignant hepatic tissues, but not in most normal adult hepatocytes [69–72]. The upregulation of it participates in the early stages of neoplastic change [73]. EpCAM plays an important role in cell proliferation, migration, and mitogenic signal transduction [74].

Recently, the EpCAM has been identified as a potential marker of LCSCs. EpCAM previously has been identified as a marker for stem/progenitor cells of mature liver and oval cells [70, 75, 76]. Yamashita et al. [77, 78] revealed that EpCAM^+^ HCC displayed the traits of LCSCs and activated Wnt/*β*-catenin pathway, whereas EpCAM^−^ HCC displayed genes with traits of mature liver cells. They also proposed a classification system which was defined by EpCAM and a-fetoprotein (AFP); meanwhile they found that EpCAM^+^AFP^+^ cells seemed to present features of LCSCs. Compared with EpCAM^−^ cells, EpCAM^+^ HCC cells had many properties, including being more tumorigenic and invasive [79]. Compared with EpCAM^−^ cells, only EpCAM^+^ cells could effectively initiate the development of invasive tumors in the case of xenografted tumor in NOD/SCID mice even after serial transplantation. Compared with CD133^+^EpCAM^−^, CD133^−^EpCAM^+^, and CD133^−^EpCAM^−^ counterparts, CD133^+^EpCAM^+^ cells possessed more representatives for TICs in HCC Huh7 cells, such as abundance in side population cells, increased colony-formation ability, higher differentiation capacity, drug resistance to some chemotherapeutics, preferential expression of stem cell-related genes, more spheroid formation of culture cells, and stronger tumorigenicity in NOD/SCID mice [80]. Recently, a research comprising 110 HCC tissues and 98 adjacent nontumor tissues by univariate and multivariate analysis revealed that patients with PTEN^−^CD133^+^ or PTEN^−^EpCAM^+^ HCC had shorter recurrence-free survival and overall survival times. The combination of PTEN with CD133 or EpCAM expression may serve as a screening tool to monitor recurrence and predict prognosis [81]. These findings suggest that EpCAM^+^ plays an important role in the beginning of HCC.

Zhang and his colleagues [82] prepared an anti-EpCAM bispecific T cell engager (BiTE) 1H8/CD3 which was derived from 1H8 (an anti-EpCAM monoclonal antibody) and was used to treat HCC in vitro and in vivo. And they found that in vivo the 1H8/CD3 absolutely inhibited the growth of Huh7 and Hep3B xenografts, which indicated that anti-EpCAM BiTE 1H8/CD3 was a promising therapeutic agent for HCC treatment. EZH2l was essential for HCC cells self-renewal and pharmacological inhibition via 3-deazaneplanocin (DNZep) to reduce the level of EpCAM^+^ HCC CSCs. Further, DNZep exhibited more powerful effects than 5-FU in terms of suppressing the quantity of EpCAM^+^ CSCs [83]. Moreover, a recent report revealed that metformin, along with impairing the capability of self-renewal of HCC cells, could reduce the number of EpCAM^+^ HCC cells [84]. Taken together, these discoveries suggest that EpCAM knockdown can be a promising therapeutic agent for HCC treatment.

### 2.7. SP Cells (ABCG2)

The side population (SP) cells were first described by Goodell et al. [85] in the murine bone marrow cells; these cells were detected for their ability to efflux Hoechst 33342 dye by an adenosine triphosphate- (ATP-) binding cassette (ABC) membrane transporter, such as MDR1 and ABCG2 [86]. It has been reported that SP cells could be purified from human HCC cell lines [87–89]. In HCC, SP cells possess CSC-like features such as high proliferative potential and antiapoptotic property and may be correlated with metastatic potential, tumorigenesis, and therapeutic resistance [87, 89, 90]. Zhou et al. [91] found that the expression of Bcrp1/ABCG2 gene determined the phenotype of SP. Further, Shimano et al. [92] revealed that hepatic oval cells, which harbored several features of stem cells, had the SP phenotype. The expression level of ABCG2/Bcrp1 mRNA was well relative to the number of oval cells. All of these data support the fact that SP cells can serve as a marker of liver CSCs.

To detect SP cells in HCC specimens in situ is not technically viable, so the examination of these ABC transporters, such as ABCG2, may help localize CSCs. ABCG2 is an ABC half-transporter which is overexpressed on the SP cell membrane and is described as the determinant for the SP cells, and the expression pattern of it markedly influences the levels of drug efflux from HCC cell lines [93]. Zen et al. [94] found that other stem cells markers such as CK19 and AFP were mainly presented on ABCG2^+^ subpopulations and that indicated ABCG2^+^ cells played a crucial role in hepatocarcinogenesis and maintenance of the cancer cell hierarchy of human HCC compared with ABCG2^−^ cells. Study of Xi et al. [95] also supported the theory that ABCG2 might be a potential marker for LCSCs. Further study explored the mechanism of ABCG2 expression in HCC, demonstrating by altering the subcellular localization of ABCG2 that the Akt pathways regulated the SP phenotype activity and the ABCG2-induced chemotherapy resistance could be resisted via inhibited Akt signaling [93, 96].

### 2.8. DLK1 (Delta-Like 1 Homolog)

DLK1 is a transmembrane and secreted protein with epidermal growth factor- (EGF-) like repeats, which is also known as preadipocyte factor 1 (Pref-1) [97]. DLK1 is a surface antigen present on hepatoblasts but not from mature hepatocytes in neonate and adult rodent liver [97]. DLK1 is highly expressed in hepatocellular, pancreas, colon, and breast carcinomas [98]. It has been serving as a molecular marker of CSCs in several hard-to-detect malignancies. Compared with DLK1^−^ cells, DLK1^+^ HCC cells show stronger ability of chemoresistance, colony-formation, spheroid colony-formation, and tumorigenicity in vivo [99]. DLK1^+^ patients have a shorter survival time than DLK1^−^ patients. A study [100] implied that DLK1 was an independent prognostic factor that the expression of it did not seem to correlate with other classic prognostic factors such as AFP, tumor-node-metastasis (TNM), and vascular invasion. Yanai et al. [98] revealed that human (h) DLK1 was expressed in human fetal but not in mature liver and that 20% of all HCCs were hDLK1^+^. They suggested that hDLK1 might be a molecular marker specific for the early stages of many of tumors, including lung cancers, HCC, pancreatic islet carcinomas, glucagonomas, and gastrinomas, which led to the development of promising diagnostic tools and therapeutic agents. The sorted DLK1^+^ HCC cells were highly chemoresistant, such as doxorubicin, cisplatin, epirubicin, and 5-FU. The suppression of endogenetic DLK1 through RNA interference could markedly inhibit cell growth, proliferation, colony-formation, spheroid colony-formation, and in vivo tumorigenicity of HepG2, Hep3B, and Huh7 cells [99, 101].

### 2.9. Nope (Neighbor of Punc E11)

Nope is a transmembrane protein belonging to the immunoglobulin (Ig) superfamily, which is thought to be indispensable in axon guidance on account of similarity with the guidance receptors such as Neogenin and deleted in colorectal cancer (Dcc) protein [102]. Nope expression was selectively detected in all analyzed HCC samples but was absent in normal liver or dysplastic lesions and at the beginning of preneoplastic lesions transforming to malignant HCC high induction of Nope expression was detected. The overexpression of Nope by hepatocytes associated with a later and more advanced stage of HCC suggested the potential of Nope as a prognostic factor. Recently, using cDNA microarray studies, Nierhoff et al. [103] identified Nope as a new cell surface marker for murine fetal hepatic stem/progenitor cells. In analogy to known oncofetal tumor markers, such as AFP and Gpc-3, Marquardt et al. [104] established Nope as a new and promising oncofetal surface marker for murine and human HCC and provided evidence for its specific expression in hepatoma cell lines and primary HCC. By quantitative real-time RT-PCR and Western blot analyses, Nope expression declined rapidly and remained barely detectable in the adult liver postnatally. Using immunohistochemical costainings for Nope- and epithelial-specific markers (E-cadherin), early hepatoblasts markers (AFP) and biliary marker proteins (CK19) revealed that Nope was initially expressed on bipotent hepatoblasts and persisted thereafter on committed hepatocytic as well as cholangiocytic progenitor cells during late fetal liver development [105]. The results indicate that Nope is a promising candidate to identify and isolate LCSCs from the adult liver.

### 2.10. DCLK1 (Doublecortin-Like Kinase 1)

DCLK1, also named as fetal antigen-1 or pG2, is a microtubule-associated CSC protein that catalyzes tubulin polymerization into microtubules. Several researches have demonstrated that DCLK1 is overexpressed in some solid tumors (colon, intestine, and pancreas) including HCC [106–109]. DCLK1 is a hepatic stem/progenitor cell marker in fetal livers which plays a vital role in oncogenesis of HCC [75, 97, 110]. Ali et al. [111] demonstrated that the influences of fluvastatin (FLV) against HCV and HCV-induced HCC through interaction with the DCLK1 microtubule axis may present a potential novel therapeutic target. Frequently lymphoid aggregates which are detected such as hepatic epithelial and stromal cells of internodular septa widely expressed DCLK1 and S100A9. They further revealed that the high expression of DCLK1 also correlated with increased levels of S100A9, c-Myc, and BRM levels in HCV/HBV-positive patients with cirrhosis and HCC. DCLK1 appeared to be a novel therapeutic target for the treatment of HCC. Silenced DCLK1 inhibits S100A9 expression and HCC cell migration [112]. Sureban et al. [113] revealed that DCLK1 was upregulated in HCC and cirrhosis controls (CCs) epithelia and stroma compared with noncirrhosis controls (NCCs), and compared with people from CC and NCC a remarkable increase in plasma DCLK1 has been observed in HCC. They found that DCLK1 was overexpressed in HCC tumors relative to adjacent normal tissues, and the high expression of DCLK1 cells had more EMT. Further, knockdown of DCLK1 on Huh7.5-derived tumor xenograft growth resulted in growth arrest and an arrested downregulation of c-Myc and EMT transcription factors ZEB1, ZEB2, Snail, and SLUG via let-7a and miR-200 miRNA-dependent mechanisms. Their findings suggest that the detection of elevated plasma DCLK1 may provide a potential companion diagnostic marker for patients with cirrhosis and HCC. Their results support the fact that DCLK1 is a biomarker for detection and a therapeutic target for eradicating HCC.

## 3. Markers in the Cytoplasm

### 3.1. OV6

Hepatic oval cells (HOCs), defined as liver stem/progenitor cells in the liver Herring pipe, are bipotential which can differentiate into hepatocytes and cholangiocytes [114, 115]. OV6 is a mouse monoclonal antibody isolated from carcinogen treated rat liver [49], which is a small cell with a property of ovoid nucleus and an underrepresented cytoplasm. OV6^+^ cells are located in the ductular proliferative cells and periseptal hepatocytes in diseased liver but not in normal human liver tissue [115]. The mature hepatocytes are unable to regenerate in severe hepatocellular necrosis, including chronic viral hepatitis and alcoholic and nonalcoholic liver disease. Activation of the potential stem cell compartment results in the formation of reactive ductules with a high level of oval cell markers. OV6 is a hepatic progenitor/stem cell marker, which has recently been identified as a putative marker for LCSCs [116, 117].

Compared with OV6^−^ cells, Yang et al. [118] reported that OV6^+^ HCC cells had a higher tumorigenic ability and resistance to standard chemotherapy in NOD/SCID mice. Moreover, activation of the Wnt pathway could increase the level of OV6^+^ cells and inhibit the *β*-catenin signaling which leads to a decrease in the proportion of OV6 cells. Furthermore, the expression of CD133^+^ was found to be observably enriched in the OV6^+^ cells, indicating that OV6^+^ was a potential LCSC marker. Using magnetic bead separation, Yang et al. also [119] isolated OV6^+^ cells from HCC cell lines SMMC7721 and Huh7 and revealed that these OV6^+^ HCC cells not only possessed a greater capacity of self-renewal and tumorigenicity in vitro and the xenograft OV6^+^ HCC cells in NOD/SCID mice but also exhibited more invasive and metastatic potentials both in vitro and in vivo. In addition, patients with high expression of OV6^+^ tumor cells were relative to aggressive clinicopathological features and poor prognosis. Furthermore, in OV6^+^ cells CXCR4 was overexpressed. The upregulation of the SDF-1 could result in increasing the population of OV6^+^ HCC cells, and by a specific CXCR4 inhibitor (AMD3100) or transfection of siRNA targeting CXCR4, the effect of SDF-1 was blocked. These results suggest that OV6^+^ HCC cells possess self-renewal capacity, tumorigenicity, and invasion, and the expression of OV6 is regulated by the Wnt/*β*-catenin and SDF-1/CXCR4 pathway within tumors.

### 3.2. ALDH (Aldehyde Dehydrogenase)

Aldehyde dehydrogenase (ALDH) is a prevalent intracellular enzyme that catalyzes the irreversible oxidation of a variety of cellular aldehydes. There are 17 isoforms of ALDH in human that also localize to the mitochondria in addition to cytosol [120]. ALDH^+^ cells can be identified by ALDEFLUOR reagent using flow cytometry or florescent microscopy. ALDH^+^ cells have been detected in cancer tissues including breast, liver, and acute myelogenous leukemia, and they are considered as CSCs based on their proliferation rates, migration, and adhesion ability. The metastatic potential of ALDH^+^ cells is higher than that of ALDH^−^ cells, and they also contribute to cancer chemoresistance and oxidative stress response [121]. Several studies had suggested that high level of ALDH estimated by flow cytometry could be a marker of liver progenitor/stem cells in normal liver [122] and CSCs in HCC [9].

The expressions of several different ALDH isoforms and ALDH enzymatic activity in liver cell lines were analyzed by Ma et al. [9]. They found that ALDH was positively correlated with the expression of CD133 by dual-color flow cytometry. They also found that the majority of ALDH^+^ HCC cells were CD133^+^, yet not all CD133^+^ HCC cells were ALDH^+^. Further studies found that CD133^+^ALDH^+^ cells were remarkably more tumorigenic than other counterparts. These data identified that ALDH, combined with CD133, could more specifically characterize the tumorigenic LCSCs. Recently, by activating the ROS-p38 MAPK pathway, Chiba et al. [123] indicated that a small molecule inhibitor of ALDH (disulfiram) had been shown to downregulate the bulk of CSC markers and suppress the self-renewal ability of HCC cells.

Furthermore, recently, the role of ALDH1A1 expressed in HCC is a hot research area. It is one of an isoform of the ALDH1 family, which synthesizes retinoic acid (RA) from the retinene and it is a crucial regulator for the RA signaling pathway [124]. RA induces gene transcription and hence regulates a wide variety of biological processes like cell proliferation, differentiation, cell cycle arrest, and apoptosis [124, 125]. Because of these characteristics of “stemness,” the ALDH1 family is considered to be a stem cell marker [126]. Compared with CD133^+^ and CD133^−^ subpopulations isolated from Huh7 and PLC8024, Ma et al. [9] identified ALDH1A1 as one of the proteins that was preferentially expressed in the CD133^+^ subfraction using a two-dimensional PAGE approach. Dollé et al. [122] revealed the same conclusion subsequently. However, the reverse results were obtained from other reports [127]. They found that the high expression of ALDH1A1 was significantly relative to low serum levels of AFP, small tumor diameter, very little lymphovascular invasion, more differentiated pathology, and good stage. The high level of ALDH1A1 showed more favorable prognosis for recurrence-free survival. These conflict results suggest that the overexpression of ALDH1A1 could appear to be differentiated cells rather than CSCs in HCC.

### 3.3. CK19

Cytokeratins are typical epithelial cell markers that exist in numerous malignant epithelial cells with increased metastatic ability and malignancy [128, 129]. Cytokeratin 19 (CK19) is a cytokeratin subtype expressed in HCC [130]. CK19 is a marker for cholangiocytes, hepatic progenitor cells (HPCs), and early hepatoblasts, which is expressed in normal human liver bile duct cells and also detected scattered in the parenchyma of cirrhotic livers and HCCs [131, 132]. CK19^+^ in HCC is a stemness marker which revealed strongest correlation with invasion, increasing tumor size, decreasing tumor differentiation, metastasis, and microvascular invasion and it is an important predictive factor for prognosis, patient survival, and tumor recurrence [130, 133–135]. In CK19^+^ HCC cells the expressions of EMT-related proteins were increased, which played a dominant role in the tumor-cell invasion process [135]. CK19^+^ HCCs were well relative to the clinical and pathological properties of tumor aggressiveness and poor prognosis [135]. Further, they also found that more than 90% of CK19^+^ HCC cells in a tissue microarray cohort expressed at least one other stemness-related marker, while EpCAM, c-kit, and CD133 were more frequently expressed alone, implying that CK19 may be more “specifically” related to stemness than other markers. Kawai et al. [136] demonstrated that CK19 was a novel CSC marker associated with EMT and TGF-*β*/Smad pathway, and these features were restrained by CK19 knockdown or treatment with a TGFbR1 inhibitor. They also found that CK19^+^ cells revealed high proliferation ability and resisted 5-fluorouracil in vitro. Using immunohistochemistry, CK19^+^ HCC patients had significantly poorer recurrence-free survival and higher tumor TGFbR1 expression compared with CK19^−^ patients. Using immunohistochemical analysis, Cogliati et al. [42] searched for stem/progenitor cells in 13 HCC and 7 CC archived specimens. They found that both liver tumors presented a higher amount of CK19 (+) HPCs.

## 4. Markers in the Nucleus

### 4.1. Nanog

The Nanog gene, a member of the homeobox family of DNA binding transcription factors, is recently identified in a screen for pluripotency promoting genes. Growing evidence has indicated that Nanog plays a crucial role in tumorigenesis. Loss-of-function studies demonstrated that Nanog was essential for amount of epithelial malignancies in part through regulation of the CSCs population [137]. Nanog played an important role in the development of solid tumors and accumulated evidence showed that the expression and levels of Nanog were upregulated in breast, ovarian, colorectal, gastric, head and neck squamous cell, hepatocellular, lung, and prostate carcinoma [138–148], suggesting that the Nanog may play a potential role in tumorigenesis. Compared with Nanog^−^ Huh7 cells, Nanog^+^ Huh7 cells possessed CSC properties, such as increased chemotherapy resistance, self-renewal, and tumor sphere formation [145]. Several studies demonstrated that Nanog was a prognostic marker for unfavorable survival in HCC. In a cohort of 59 HCC patients, the high expression of Nanog protein was relative to poorer overall and disease-free survival [145]. In Nanog^+^ Huh7 HCC cells, the self-renewal and Nanog expression were decreased by the IGF1R inhibitors, such as picropodophyllin and AEW541, which suggested that the insulin growth factor (IGF) pathway might be downstream of Nanog [145]. These results suggest that there exist a cross-talk mechanism between Nanog and IGF1R to maintain the CSC population in HCC. By activating the Nodal/Smad3 signaling, Nanog in HCC cells was reported to promote EMT and cell invasion. The high expression of Nanog in low Nanog-expressing Huh7 HCC cells led to an increase in cell invasion and induced EMT with the hallmark high vimentin/low E-cadherin signature [149]. Using the HepG2 cell line, Zhou et al. [150] demonstrated that HCV core protein inducing Nanog expression was followed by enforcing expression of phosphorylated STAT3 protein and was attenuated by suppressing STAT3 phosphorylation. Recently, several researches by Yin et al. [151, 152] indicated that the coexpression of Oct4 and Nanog was dramatically associated with recurrence, metastasis, prognosis, and drug resistance of HCC. Following molecular mechanism investigation revealed Oct4/Nanog-regulated EMT change through STAT3-dependent Snail activation. In addition, the knockdown of STAT3 abrogated the change and invasion/metastasis of Oct4/Nanog-mediated EMT in HCC. These findings promote a novel therapeutic target for treating the progression and metastasis of HCC with CSC-like signatures and EMT phenotype.

### 4.2. SOX9

SOX9 (sex determining region Y [SRY] related high mobility group box 9) is a member of the SRY box gene superfamily [153] and belongs to the subgroup of SOX E genes, which plays critical roles in the regulation of differentiation of astrocytes, oligodendrocytes, and Schwann cells [154]. SOX9 has also been proved to be a protooncogene in a number of malignancies, such as breast, prostate, bladder, ovarian, intestinal, and gastric tumor [155–161]. Through upregulation of Oct4, it is involved in maintenance of cancer stem-like properties including self-renewal, chemoresistance, and tumorigenicity [162]. Guo et al. [163] first analyzed SOX9 protein and mRNA expression in human HCC tissue and found that it was associated with patient clinical outcome. The overexpression of SOX9 was observed in tumor tissues with higher tumor stage, poorer disease-free survival, and poorer overall survival of HCC patients. Their further studies [164] suggested that SOX9 directed regulator miR-101 was the potential tumor suppressor miRNA for HCC. They discovered that the level of SOX9 was downregulated by the proposed target miRNA, miR-101, which was also confirmed by the luciferase reporter assay, suggesting that miRNA-101 directly targets SOX9 in HCC tissues. And the enforced expression of miR-101 and suppression of SOX9 by siRNA inhibited not only the cell proliferation but also tumorigenicity of HCC cell line in vitro. Recently, it is assumed as a hepatic stem/progenitor cell. But what role they may play in the development of HCC is still unclear. Further researches are needed to validate this point.

## 5. Others

### 5.1. SALL4 (Sal-Like Protein 4)

SALL4, the human homolog of the Drosophila spalt homeotic gene, is a member of the family of zinc finger transcription factors which regulates pluripotency, organogenesis, and self-renewal in embryonic stem cells (ESCs) [165]. SALL4 is crucial for maintaining stemness features of ESCs through both transcriptional and epigenetic controls including direct interaction with Oct4, SOX2, and Nanog [166]. SALL4 expression is detected in plasma cell myeloma and acute lymphoblastic leukemia, as well as in solid tumors such as breast, lung, and colorectal cancer [167–170]. Using immunohistochemistry, reports [171, 172] revealed that SALL4 was expressed in the nucleus and cytoplasm. In the liver, a high expression of SALL4 was detected in normal human hepatic stem cells, hepatoblasts, and biliary tree stem cells but not in mature hepatocytes, and it played a dominant role in hepatic cell-lineage commitment [173]. Several studies revealed that SALL4 mediation increased the expansion of human hematopoietic stem and progenitor cell (HSC/HPC), likely by increasing self-renewal activity and inhibiting differentiation processes. Yong et al. [174] hypothesized that SALL4 could be an oncofetal marker and an attractive therapeutic target in HCC with progenitor cell origin. SALL4 was an indicator of stem cells and a prognostic marker in hepatocarcinogenesis, relative to cell and tumor growth, with chemoresistance to 5-FU, and its suppression resulted in differentiation and slowed tumor growth [171]. The overexpression of SALL4 of hepatocytes was correlated with a more aggressive subtype of HCC and poor prognosis, such as tumor recurrence, tumor size, multiplicity, vascular invasion, pathological tumor stage, and overall survival rate [137, 171, 172, 174]. The expression of SALL4 was positively correlated with EpCAM but not with the level of CK19 [169]. Moreover, the coexpression of SALL4 and EpCAM was associated with significantly decreased overall survival. Recently, SALL4 is a novel therapeutic target for HCC. Using short hairpin RNA (shRNA) revealed that the knockdown of SALL4 in HCC cells resulted in a decreased tumorigenicity, increased apoptosis, and a more hepatocyte-like gene expression signature in human hepatoma cell lines. The PTEN-AKT pathway was involved in SALL4-induced hepatocarcinogenesis. The tumor suppressor PTEN has been shown to be repressed among the target genes by SALL4 [171]. Using a special inhibitory SALL4 peptide (12 amino acids in length) blocked the effects of SALL4. Zeng et al. [172] found that the SALL4 expression status was relative to histone deacetylase activity in cell lines, such as KRT19, EpCAM, and CD44, and the histone deacetylase inhibitor successfully suppressed the proliferation of SALL4^+^ HCC cells. These studies indicate that SALL4 is a valuable biomarker and therapeutic target for diagnosis and treatment of HCC with stem cell properties.

In conclusion, further studies are needed to determine the association of LCSC markers as well as pathologic properties with the clinical outcome of HCC. Because of the great heterogeneity of HCC, the predictive potential of a single marker is limited to a very small subpopulation. In this review, we found that the coexpression of CD44^+^CD133^+^, CD44^+^CD90^+^, CD133^+^EpCAM^+^, ALDH^+^CD133^+^, or SALL4^+^EpCAM^+^ could be better defined in LCSCs. The double-positive for these markers displayed a more aggressive phenotype (such as extensive proliferation, self-renewal, and spheroid formation of cancer cells), formed metastatic lesions, and resisted multiple chemotherapeutics. So the combination of several LCSC markers may provide higher specificity and reliability way to detect HCC. The identification of LCSCs plays a dominant role in the targeted therapy and prevention of chemoresistance and metastasis in HCC will provide a novel method for the treatment of HCC in the future.
